# Assessing disparities in air conditioning operation across Southern California

**DOI:** 10.21203/rs.3.rs-10103387/v1

**Published:** 2026-07-01

**Authors:** Diego Ramos Aguilera, Yishi Wang, Lu Zhang, Jiachen Zhang, Kelly T. Sanders

**Affiliations:** a Dept. of Civil and Environmental Engineering, University of Southern California, Los Angeles, CA, USA; b Dept. of Population and Public Health Sciences, University of Southern California, Los Angeles, CA, USA

**Keywords:** air conditioning use, adaptive capacity, urban heat, environmental justice, inequity

## Abstract

We employed smart-meter data from 140,000 households to examine how residential AC operation in Southern California was associated with meteorological conditions, socioeconomic characteristics, and housing-related factors across different climate zones. We characterized AC operation patterns through two metrics that quantify the daily duration and intensity of cooling, enabling an assessment of adaptive capacity to heat beyond what traditional measures of AC ownership capture. Our results quantify how socioeconomic status and dwelling characteristics shape cooling behavior, including the likelihood, duration, and electricity consumption of AC operation, both during daytime and nighttime. After normalizing cooling metrics to account for heat exposure and dwelling size, we examined potential cooling gaps across our study region, relating these to income disparities and to the presence of vulnerable populations. Our work highlights the need for equity-oriented adaptation strategies that reduce the cost burden of cooling in order to mitigate heat-related risks.

## 1. Introduction

Populations around the world are increasingly facing the threat of extreme heat as the planet warms and local climates are disrupted. Evidence from atmospheric studies shows that the world is experiencing not only a gradual upward shift in average temperatures, but also an even faster rise in temperature extremes, driving unprecedented heat events [1]. Many regions already show evidence that extreme heat events are becoming more frequent, longer lasting, and more intense [2]. Beyond certain physiological limits of heat tolerance, human comfort and survivability are threatened, making cooling solutions a critical necessity for the population [3, 4]. When unmitigated, extreme heat can have numerous impacts, ranging from direct impacts on health [5] to stressing social, infrastructural, and natural systems [6].

Air conditioning (AC) has become established in the United States and in many parts of the world as the default residential cooling technology, allowing the indoor thermal environment to be modified to achieve comfortable conditions. Unlike other solutions such as the use of natural ventilation or ceiling fans, it can provide relief in extreme heat conditions [7, 8]. There is ample evidence of the protective effect of AC, with AC prevalence being associated with reduced mortality and morbidity [9, 10, 11, 12]. However, the high energy intensity of AC technologies may exacerbate grid stress during periods of high power demand and raise concerns about power-sector emissions [13, 14, 15]. Grid reliability is also an important consideration, as power outages can have serious public health consequences for populations depending on AC to manage otherwise unbearable temperatures [16]. Moreover, the high energy requirements of AC may constitute a barrier to its use [17, 18]. Qualitative studies confirm that the cost of electricity is a deterrent for AC use in low income or heavily burdened households [19, 20]. Forgoing cooling at times of extreme heat can lead to heat-related illness, the aggravation of pre-existing health conditions, and even mortality [21]. Therefore, determining where and when such energy limiting behavior may exist, and the conditions that may be associated with it, remains a pressing public health question.

To date, most research examining gaps in cooling access has relied on measures of AC ownership, a binary variable describing whether a household owns an AC system. Such studies have documented disparities in AC ownership across income levels at both national [22, 23] and city scales [24, 25], and also reported lower ownership by marginalized groups [26] and renting populations [27]. Similarly, AC owners have higher energy use intensity than their non-AC-owning counterparts [28], and lower-income households tend to occupy less energy-efficient dwellings [29]. Despite the recent interest in improving estimates of AC ownership (e.g., [30, 31, 32]) there is an evident shortcoming to this body of work: having access to AC does not imply using it [28]. Furthermore, Doremus et al. [17] suggest that in countries with generalized high AC ownership like the U.S., it becomes especially important to consider questions of AC operation. Such metrics may reflect *when* AC is used, for *how long*, or *how intensely* it is run.

The recent proliferation of smart-meter electricity data has enabled the bottom-up modeling of AC (e.g., 33, 34, 35), providing an opportunity to explore patterns of AC operation. For instance, Elmallah et al. [36] employed smart-meter data to relate the likelihood of a household having AC to demographic, housing, and climate characteristics. Their approach characterized disparities in access to cooling by comparing projected cooling needs to observed AC access. Cong et al. [37] found differences in the outdoor temperature at which households turn on their AC systems across income groups. These differences, which they describe as a “cooling equity gap”, constitute evidence of energy limiting behavior (see also Huang et al. [38]). Recently, Peplinski et al. [39] created an algorithm to identify cooling and heating hours from hourly smart-meter data, based on electricity-temperature relationships. Despite these advances, few studies have attempted to explicitly determine how meteorological, sociodemographic, and housing-related factors affect the duration of AC use, and the electricity loads associated with it. Addressing this knowledge gap is important because it can provide a novel way of understanding disparities in AC use across the population.

In this work, we examine how weather, sociodemographic, and housing-related factors are associated to the operation of AC and examine whether structural disparities exist across the population. We focus on five counties in Southern California, a geographically and demographically diverse region and home to one of the largest metropolitan areas in the United States. Estimates of AC ownership in the region reveal that on average 79% of the population had access to AC between the years 2015–2019 [39]. California’s AC ownership remains lower than the national average, which is estimated at 88% [40]. This lower adoption of AC in California is due in part to the state’s mild climates found along the coast, where a large proportion of the population lives. However, in recent decades annual cooling degree days (which compare average daily temperatures to a base of 65°F, or 18.3°C) have been steadily increasing across California, elevating the need for cooling to mitigate indoor heat exposure [41]. The spatial heterogeneity in AC adoption, demographics, and climates make Southern California a valuable case study.

Specifically, we set out to address the following research questions:

1. How does an additional degree of temperature affect the duration of AC usage and the electricity intensity? Is this relationship consistent across different climates and for different population groups?
2. How does the built environment (e.g., dwelling size, age, or value) shape patterns of AC operation?
3. Do income-constrained or vulnerable groups employ AC differently in terms of cooling durationor electricity consumption?

To this end, we create two metrics of household-level AC operation, based on the dataset of hourly AC operation created by Peplinski et al. [39] for about 140,000 households in Southern California. The first is a measure of daily duration (measured in hours) of AC use (henceforth, “cooling hours”), and the second is a measure of the hourly electricity expenditure associated with AC use (“cooling load”, measured in kilowatt-hours, kWh). We refer to these two metrics jointly as “AC operation”. We combine these data with census tract-level sociodemographic information, individual housing information from assessors’ databases, and gridded hourly meteorological data. Our analysis is two-fold: first we employ logistic and ordinary least squares regression to test the statistical association between AC operation and weather, sociodemographic, and housing-related characteristics. Then, after normalizing for the heat experienced in each location and for house size, we compare AC operation across the population and determine whether a potential cooling gap exists. By linking smart-meter data to socioeconomic and housing characteristics, our work advances our understanding of cooling equity and offers an empirical basis for targeting interventions that strengthen adaptive capacity under extreme heat.

## 2. Results

### 2.1. Descriptive analysis of cooling use in Southern California

**Figure 1:**
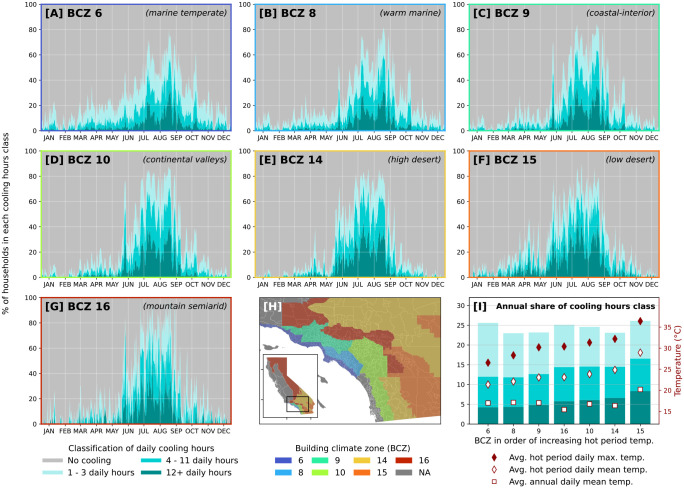
(*a-g*) Percentage of households in each day of 2019 using no cooling (grey), mild cooling (1–3 hours; light blue), intense cooling (4–11 hours; medium blue), and very intense cooling (12 or more hours; dark blue), by Building Climate Zone (BCZ). (*h*) Map depicting the locations of BCZs in Southern California (inset showing location within California). NA = BCZ not in our dataset. (*i*) Annual share of cooling hours class for each BCZ, with overlaid points indicating average hot period maximum and mean temperatures and average annual daily mean temperatures.

Our results show that most of the cooling demand in Southern California (~77% of cooling hours) occurred between mid-June and mid-October. We define this four-month interval as the ‘hot period’. To characterize cooling behavior, we classified each household-day by the number of daily cooling hours and found substantial variation across building climate zones (BCZs) (Figure 1). In coastal regions, households employed fewer cooling hours per day but operated AC over a larger fraction of the year than hotter inland regions. For instance, households in BCZ 6 (characterized by a temperate marine climate) showed a greater prevalence of ‘mild’ cooling days (using AC 1–3 hours daily) across the year than inland regions, reflecting moderate but persistent cooling needs associated with relatively mild temperatures and a narrow diurnal temperature range (Figure 1a). In contrast to coastal climates, households in continental valleys (BCZ 10), desert regions (BCZ 14 and 15), and mountainous regions (BCZ 16) displayed a larger share of ‘intense’ (up to 11 daily hours) or ‘very intense’ (more than 12 hours) cooling days concentrated during the hot period (Figure 1d–g). This cooling pattern was likely driven by the extreme temperatures that occur during these months (Figure 1i). With respect to cooling load, households in BCZ 10, 14, 15, and 16 exhibited loads that were, on average, 37% greater per cooling hour than those of households in BCZ 6 and 8 (Table 1). In addition, cooling loads were on average 2.4 times greater than non-cooling loads. For reference, the locations of BCZs are shown in Figure 1h.

**Figure 2:**
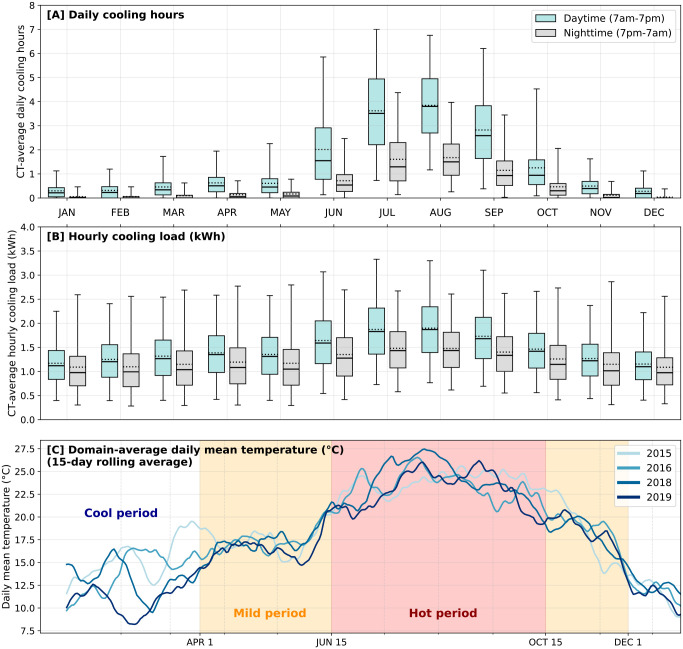
Monthly variation of household (*a*) daily cooling hours and (*b*) average hourly cooling load, averaged at the census tract (CT)-level. The light blue shade indicates daytime hours (7 am - 7 pm) and gray shade indicates nighttime hours (7 pm - 7 am). Boxplot whiskers represent the 95% percentile interval. Medians are shown with solid lines and means with dotted lines. (*c*) Domain-average 15-day rolling average of daily mean temperature. Color shading indicates our definition ‘cool’ (no shading), ‘mild’ (orange), and ‘hot’ (red) periods employed in this study.

Beyond the regional differences described above, domain-wide AC operation revealed a clear temporal structure, both seasonally and diurnally (Figure 2a–b). During the peak cooling months of July and August, census tracts cooled for an average of 3.7 daytime hours (out of 12) and 1.6 nighttime hours. Average hourly cooling loads were highest during the summer months of June through August, suggesting that AC systems operated for a larger fraction of each hour compared to a more sporadic use outside of the summer season. Across the year, cooling loads were consistently higher during daytime than nighttime, with differences reaching up to 0.4 kWh per hour during the hot period. This pattern likely reflects both longer AC duty cycles during daytime and the lower efficiency of AC operation at higher outdoor temperatures. The daytime-nighttime contrast was more pronounced in inland climate zones (Table 1), likely because of larger diurnal temperature swings. When we accounted for differences in house size, these disparities remained: households in inland regions employed 27% more cooling electricity than their coastal counterparts (Table 2).

**Figure 3:**
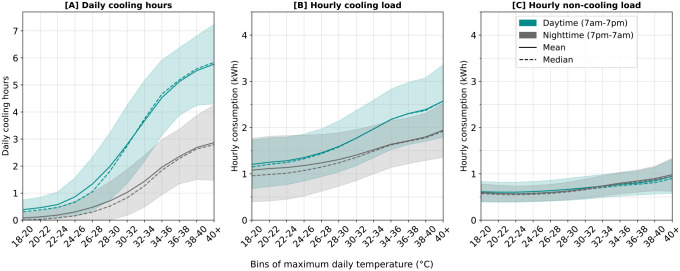
Variation in cooling and electricity usage across bins of maximum daily temperature: (*a*) daily cooling hours, (*b*) average hourly cooling load, and (*c*) average hourly non-cooling load. The blue line represents daytime hours (7 am - 7 pm) and gray represents nighttime hours (7 pm - 7 am). The solid lines represent the mean, the dashed lines represent the median, and the shaded regions indicate the standard deviation.

The spatiotemporal patterns identified above are partly driven by outdoor temperatures. Figure 3 shows the variation of daily cooling hours, average hourly cooling load, and average hourly non-cooling load across bins of maximum daily temperature. In days with maximum temperature higher than ~23°C, the number of daytime cooling hours increased steeply with temperature, up to about 35°C, when households were cooling for an average of 4.5 hours during daytime and 2 hours during nighttime. Beyond this point, hotter days resulted in less additional cooling hours. In terms of cooling load, we observed a more consistent relationship with maximum daily temperature, with only a slight change in gradient after 35°C (Figure 3b). Differences in cooling load between daytime and nighttime were smaller than for cooling hours. Figure 3c shows a small increase in hourly non-cooling load for hotter days, although this increase may be non-negligible in relative terms. This effect may be due in part to the attribution of cooling hours as non-cooling by our model when only a weak temperature dependence is found, or to the fact that people may seek to be indoors more in the hottest days, therefore making more use of household appliances and increasing the power draw during those hours.

### 2.2. Statistical association of cooling use with meteorological, sociodemographic, and housing-related factors

**Figure 4:**
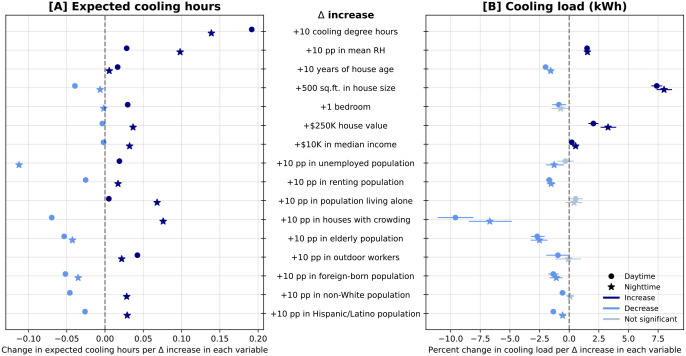
Relationships between cooling and different environmental, sociodemographic, and housing-related characteristics. (*a*) Average discrete changes (ADC) for cooling hours, showing the change in cooling use for a given delta in covariates, and (*b*) percentage changes in cooling load for a given delta in covariates. Daytime hours are represented with circles and nighttime hours with triangles, and lines are used to represent the 95% confidence interval in (*a*) expected cooling hours and (*b*) the regression coefficients of cooling load. The color indicates significance (dark blue if positive and significant, light blue if negative and significant, and gray if not significant at the *p* < 0.05 level). Note: “pp” = percentage points.

The results of the statistical models confirmed that meteorological conditions were tightly linked with AC operation. We tested models including either the maximum daily temperature or cooling degree-hours (CDH) above 20.5°C as the temperature term, and found that CDH had slightly better performance (higher R^2^, lower information criterion values) in the regression of cooling load, as well as in the ordered logit model employed for cooling hours (see Section SI.3). However, maximum daily temperature had a slightly better fit in modeling the non-cooling load and in the binary logit model. In other words, peak temperatures were more predictive of AC activation, whereas usage duration and the cooling load were more strongly associated with the cumulative heat experienced throughout the day. Given that differences were small between models, here we focus on the results of CDH models. Holding all other variables constant, an increase of 10 CDH in a day was associated with an increased likelihood of cooling by 2.4 percentage points (pp) during daytime (3.1 pp during nighttime), as well as with an increase of 0.15 hours during daytime (0.10 hours during nighttime) in duration of use (see Section SI.4). Higher CDH were also associated with increased cooling loads across all climate zones (Figure S1). Similarly, a 10 pp increase in relative humidity was associated with a longer duration of use (0.13 hours during daytime and 0.17 during nighttime) and ~2% increase in cooling load, on average (Figure 4b). When the effects of increased likelihood and increased cooling duration were combined, a 10 CDH increase resulted in 0.19 and 0.14 additional cooling hours for daytime and nighttime, respectively (Figure 4a). For reference, the average cooling hours year-round for our selected dataset for statistical analysis (note that this is different from our full sample; see Section 4.2) were 1.45 hours during daytime and 0.48 during nighttime, so these changes represent increases of 13 and 29%. In terms of relative humidity, a 10 pp increase resulted in 2% (20%) higher cooling hours on average during daytime (nighttime).

Variables relating to lower socioeconomic status of census tracts were associated with reduced cooling loads, all else being equal (Figure 4b). For example, 10 pp increases in census tract unemployment, renting population, foreign-born population, elderly population, outdoor workers, and ’crowding‘ (representing homes with more than 1.5 occupants per room) were all associated with cooling load reductions during either daytime, nighttime, or both. The scale of these changes is not directly comparable across variables: a 10 pp increase in crowding is much less likely than in renting, given their statistical distributions (see Table S1). On the other hand, higher median income was associated with increased cooling loads (about 0.2–0.5% per each additional $10,000). We tested the interaction between income and the CDH spline of the OLS model and confirmed that the association between temperature and cooling was modified by income (daytime: *F*(3, 74, 700) = 62.5, *p* < 0.001; night-time: *F*(3, 69, 900) = 3.8, *p* = 0.0098), with higher-income neighborhoods generally exhibiting steeper temperature-cooling response curves (Figure S2). In terms of demographics, a 10 pp increase in non-White and Hispanic/Latino population was associated with 0.6% and 1.3% reductions in daytime cooling load, respectively.

Cooling hours showed less consistent effects among the socioeconomic variables examined and between daytime and nighttime (Figure 4a). Generally, lower socioeconomic status was associated with reduced cooling hours during daytime but rarely during nighttime. A notable exception was unemployment: an additional 10 pp in unemployed population —who are more likely to be at home during the central hours of the day— was associated with 0.02 more cooling hours during daytime and 0.11 less cooling hours during nighttime (representing changes of +1.4% and −23%, respectively). Lower incomes were associated with fewer nighttime cooling hours, likely a result of cost concerns. Meanwhile, census tracts with higher proportions of population living alone and outdoor workers were associated with increased cooling hours during both daytime and nighttime.

In terms of housing characteristics, house age was associated with increased cooling hours, whereas larger homes were associated with fewer cooling hours, all else being equal. The opposite was true in terms of cooling load: older homes were associated with lower electricity employed for cooling, and house size had a strong, positive effect on cooling load: an additional 500 square feet was associated with daytime and nighttime cooling loads about 7.5% larger on average. This was expected given the additional volume of space that needs to be cooled in larger homes. More expensive homes were also associated with increased cooling loads, as well as increased cooling hours during nighttime, similarly to the relationships observed for census tract median income.

### 2.3. Inequity in cooling operation

**Figure 5:**
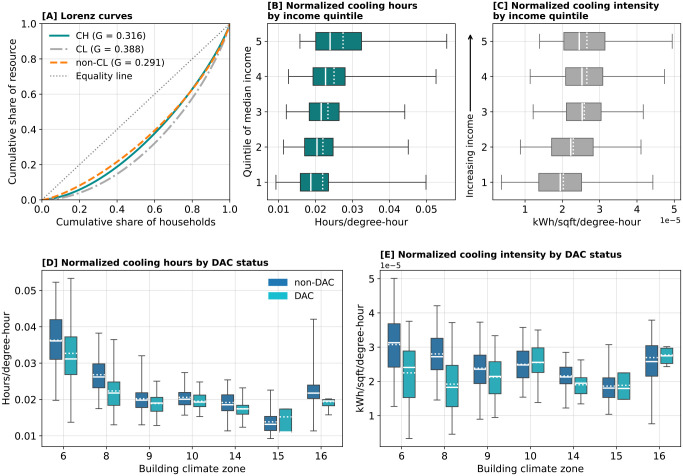
(*a*) Lorenz curves showing the level of inequality for cooling hours (CH), cooling load (CL), and non-cooling load (non-CL). Gini coefficients (G) are provided in the legend; (*b-c*) Differences in normalized cooling hours and normalized cooling intensity, respectively, across quintiles of median income, from lowest (1) to highest (5); (*d-e*) Differences in normalized cooling hours and normalized cooling intensity for disadvantaged communities (DAC) and non-DAC tracts, by building climate zone. Boxplot whiskers indicate the 95% percentile interval, medians are shown with solid lines, and means with dotted lines.

We found that the degree of inequality in cooling load across households was higher than inequality in non-cooling load and similar to the inequality in median income. As shown in Figure 5a, we quantified the degree of inequality in absolute cooling metrics (e.g., prior to normalization) and income by drawing Lorenz curves, which compare the cumulative share of each measure to the cumulative share of population (see Section 4.4). Gini coefficients (*G*) were computed to determine the overall degree of inequality, where higher values of *G* imply higher inequality. Across all BCZ, cooling load was the most inequitably distributed metric (*G* = 0.39), followed by cooling hours (*G* = 0.32), and then by non-cooling load (*G* = 0.29; see Figure 5a). To compare the degree of cooling inequality to that of median income, a census tract (CT)-level variable, we computed population-weighted versions of the Lorenz curves at the CT-level (Figure S3). Across census tracts, cooling load (*G* = 0.21) was similarly distributed to median income (*G* = 0.22), and both were more unequally distributed than cooling degree-hours, (*G* = 0.17). Meanwhile, cooling hours (*G* = 0.11) and non-cooling load (*G* = 0.13) were more equally distributed across CTs. When we investigated each climate zone individually, we also noted that for both cooling metrics, the highest inequality occurred in coastal regions (BCZ 6 and 8), compared to non-cooling load, for which the highest inequality occurred in BCZ 15 (Figure S4).

Because observed differences in cooling could result from differences in heat exposure, and the cooling load could be heavily influenced by house size, we accounted for these effects by normalizing both cooling metrics. First, we divided cooling load by the square footage of the conditioned space, thereby obtaining a measure of cooling intensity (units of kWh/sqft). Then, we divided the cooling hours and cooling intensity metrics by the daily cooling degree-hours (CDH) above 20.5°C, to quantify the cumulative heat burden throughout each day at each location. These normalized metrics, ‘normalized cooling hours’ (NCH) and ‘normalized cooling intensity’ (NCI), allow for comparisons of cooling use that are independent of dwelling size and of local heat burden. We found that higher income quintiles had higher NCH and NCI (Figure 5b–c). In both cases, the lowest income quintile exhibited lower cooling use than the remaining quintiles (NCH: Mann-Whitney *U* = 293,153, *n*_1_ = 455, *n*_2_ = 1,818, *p* < 0.001; NCI: Mann-Whitney *U* = 273,012, *n*_1_ = 455, *n*_2_ = 1,817, *p* < 0.001). NCH and NCI also exhibited weak positive monotonic relationships with income quintile (Spearman’s *ρ* = 0.286, *n* = 2,273 and *ρ* = 0.243, *n* = 2,272, respectively; both *p* < 0.001).

In line with the above findings, we found that census tracts labeled as ‘disadvantaged’ by local authorities (henceforth, DACs; see Section 4.4) —which are in many cases also low-income tracts— cooled predominantly less than non-DACs (Figure 5d–e). This difference was most evident in coastal regions (BCZ 6 and 8), but also visible in some inland climates (particularly in terms of NCI). Inland climate zones also cooled more sparingly compared to coastal regions when the heat burden was accounted for. This difference likely resulted from coastal climates making a more consistent use of AC throughout the year (see Figure 1), possibly as a way of dehumidifying indoor environments. The lower normalized cooling by disadvantaged populations may be evidence of energy-limiting behavior, by allowing indoor temperatures to rise further before turning AC systems on (lower NCI), or by limiting the use of AC for part of the day (fewer NCH). In addition to examining income and DAC status, we compared the racial and ethnic composition of high and low cooling groups (Figure S5). We found that the lower quintiles of NCH and (especially) NCI had higher proportions of Hispanic/Latino population. Differences in the demographic composition between the lowest cooling quintile and the remaining tracts were statistically significant (Table SI.9).

**Figure 6:**
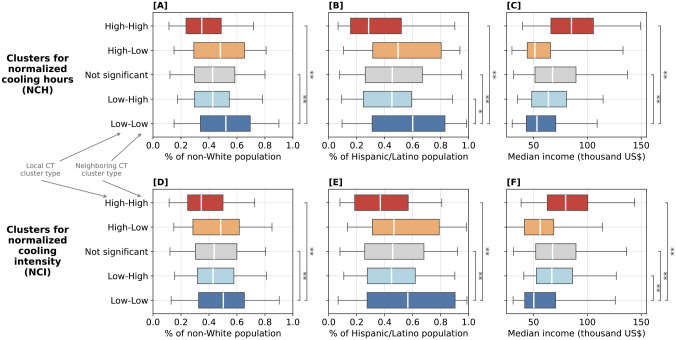
Racial, ethnic and economic composition of LISA clusters for (*a-c*) normalized cooling hours and (*d-f*) normalized cooling intensity. The first column shows the percentage of non-White population in each cluster, the second column shows the percentage of Hispanic/Latino population, and the third column shows median income in thousands of US$. Boxplot whiskers represent the 95% percentile. Results of the Mann-Whitney’s U-test between *Low-Low* cluster and others are shown with asterisks: * for *p* < 0.01, ** for *p* < 0.001.

Finally, we investigated spatial clusters and outliers to detect census tracts that were under- or over-cooling (in terms of NCH and NCI) relative to their neighbors. Figure 6a–b shows that *Low-Low* clusters of NCH (i.e., locations that have significantly low NCH and whose spatial neighbors also have significantly low NCH; see Section 4.4) had higher proportions of non-White and Hispanic/Latino population (with medians of 52.1% and 61.0%, respectively) than the other clusters (medians of 41.7% and 42.8%, respectively). A similar pattern was observed for *Low-Low* clusters of NCI (Figure 6d–e). We also noted wealth disparities across clusters: the *Low-Low* clusters for NCH and NCI had lower median income (~US$17k and 19k less, respectively) than the rest of clusters (Figure 6c,f). The above results were all significant (Table SI.10). After the *Low-Low* census tracts, the cluster with the highest proportion of Hispanic/Latino and non-White populations was *High-Low*. These census tracts represented spatial outliers with high normalized cooling in regions with generally low AC operation.

In some regions, we observed a stark difference in the predominant clusters between DAcs and non-DACs (Figures S6, S7). For instance, in BCZ 8, 28% of census tracts in DACs were *Low-Low* clusters for NCH and 36% for NCI, compared to 7% and 5%, respectively, in non-DACs. In other words, low cooling clusters were more frequently found in tracts classed as disadvantaged. The converse was true: in BCZ 6, 8, 9 and 10, *High-High* clusters of NCH were more common in non-DACs. These differences between DACs and non-DACs, which were larger in coastal climates, add to the evidence of cooling inequity across Southern California.

## 3. Discussion

Our results show that residential cooling operation has a marked spatiotemporal dependence that is tightly linked to meteorological conditions and modulated by characteristics of the built environment and of the sociodemographic context. Most cooling in Southern California over the study period occurred between June and September, which aligns with previous findings [20]. The daily duration of cooling increased nonlinearly with maximum daily temperature, with an inflection point near 35°C. Above this threshold, each additional degree resulted in progressively smaller increases in cooling hours. We hypothesize that this effect may be due to a number of reasons. Firstly, cooling saturation may be taking place, with households entirely meeting their cooling needs in the hottest days with around 6.5 hours of cooling. Secondly, this effect could result from occupancy patterns, with residents of working age and children likely spending much of the day outside of the house. Lastly, some households may be limiting their use due to cost concerns. We also found that cooling load —likely a proxy for the duty cycle of AC— increased consistently with maximum daily temperature. The quasi-linear relationship may indicate that ACs are rarely operating for the full duration of an hour, and thus that AC units are in general adequately sized. However, the increased inefficiency of cooling at hotter temperatures may also be playing a role in this relationship.

While maximum daily temperature was a stronger predictor of the decision to activate AC, using a cumulative measure of heat (CDH) was more closely associated with the duration of cooling use once activated, and with the hourly cooling electricity consumption, by capturing the total thermal load that AC needs to dissipate. The different insights provided by each temperature metric underscores the importance of separately characterizing the decision to activate the AC versus its continued operation, confirming that the two-stage structure of our hurdle logit model was appropriate for modeling cooling hours. It also points to the importance of employing metrics of cumulative heat (e.g., Nairn and Fawcett [42]), when considering the population’s cooling needs. Furthermore, we found that more humid days were associated with higher expected AC hours, supporting the increasing body of work calling for the inclusion of humidity in analyses of the population’s experience of extreme heat [43, 44].

Beyond meteorology, our results indicate that AC operation is also intricately associated with sociodemographic factors and dwelling characteristics. In general, more vulnerable census tracts (e.g., tracts with higher levels of unemployment and renting, or higher presence of foreign-born and minority populations, or those labeled as DACs) were associated with lower cooling loads and cooling hours, particularly during the daytime. Also, larger, newer, or more expensive homes were associated with increased cooling loads. Therefore, the affordability or running costs of AC are likely important factors driving cooling patterns. These associations (particularly the daytime effects) are broadly aligned with the relationships found in Northern California between demographic factors and the likelihood of households employing cooling [36]. While cooling load changes were consistent between daytime and nighttime, we found much more heterogeneity in cooling hours, possibly in response to different occupancy patterns during daytime and nighttime. Given the magnitude of nighttime effects on cooling hours, the study of nighttime cooling is particularly important.

Our results reveal an apparent economic paradox: census tracts with higher incomes and more expensive homes were associated with increased cooling loads but fewer daytime cooling hours, all else being equal. This pattern is likely due to cooling load and cooling duration capturing different dimensions of cooling behavior. The number of daytime cooling hours likely reflects the extent of cooling needs in a day. We propose that the reduced daytime cooling hours in more affluent areas may be partly explained by geographical and structural advantages. Firstly, more expensive homes are often located in cooler urban microclimates, where the presence of vegetation enhances evaporative cooling and provides shade [45, 46, 47]. Secondly, higher-quality building envelopes and better insulation reduce infiltration, thereby lowering cooling requirements and shortening cooling periods. On the other hand, the increased cooling loads by higher-income tracts aligns with the findings of Kwon et al. [18], who reported steeper cooling slopes among wealthier populations. These results suggest that efficiency gains associated with newer housing stock and improved building performance (e.g., Chen et al. [29]) are likely being outweighed by the greater cooling intensity of affluent households. A possible explanation for this is that higher-income households face lower financial constraints on AC use compared to lower-income population, who may be faced with the decision to reduce or forgo cooling during some periods of the day. This could constitute a “cooling gap” in terms of cooling load, similar to that reported by Huang et al. [38] in Arizona.

A key objective of this study was to determine whether cooling inequality existed across the region and, if so, whether vulnerable groups were systematically undercooling. Lorenz curves showed that cooling loads were more unequally distributed than the experienced heat burden (measured by CDH), and that the magnitude of this inequality was similar to that of median income. When we accounted for heat burden and stratified by BCZ, we found disparities in our cooling metrics that aligned with the economic and minority status of the population. Namely, census tracts with lower NCH and NCI tended to have a higher proportion of Hispanic/Latino population and lower median income. In addition, *Low-Low* clusters of both cooling metrics were also more frequently found within the boundaries of disadvantaged communities (DACs), which represent environmentally or economically burdened locations, as defined by the California Environmental Protection Agency [48]. Such lower cooling by vulnerable groups may constitute further evidence of potential cooling gaps existing in the region.

Finally, we observed that cooling disparities between DACs and non-DACs were particularly relevant in coastal climates. We explain this difference by theorizing that AC operation may respond to two different drivers: seeking comfort and protecting health. In the former case, vulnerable populations may be able or willing to forgo comfort for a few hours, or reduce their activity levels [4], especially when outdoor temperatures are not too elevated or nighttime temperatures are expected to bring about relief. This could lead to lower normalized cooling consumption in coastal regions with milder temperatures and ocean breezes. Instead, where cooling is critical for protecting health, it may be possible for income-constrained populations to forgo cooling, and alternatively may choose to go into debt or reduce other household expenses [17, 20]. In these cases, we would not observe lower cooling consumption by vulnerable populations.

### 3.1. Limitations and future research

Although the cooling gaps uncovered here may result from economic barriers, they could also be due to other factors, such as differences in perception of heat or thermal comfort preferences, alternative cooling practices (e.g., the use of ventilation or fans), or different occupancy schedules. In addition, habits, cultural factors (including clothing), and concerns about energy efficiency may govern these decisions to operate AC [49, 50], confounding the relationships we observed. Additionally, this work does not consider characteristics of the built environment (e.g., level of insulation, type of glazing, orientation, neighborhood tree canopy), which modulate how outdoor meteorological conditions are translated into indoor temperatures. We used building climate zones (BCZ) as a proxy for different structural dwelling characteristics [51], but this oversimplifies the heterogeneity of designs, materials, and thermal efficiency of houses within each region. Given the above reasons, we are cautious in considering the cooling disparities that we find as *potential* cooling gaps instead of as definitive evidence of energy limiting behavior. Future research should incorporate more data on personal preferences, alternative adaptive strategies, daily routines, and additional dwelling characteristics.

We also note that our estimations of AC operation are derived from hourly smart-meter data, based on a change point model of the relationship between temperature and electricity [39]. While this statistical approach has been frequently used in the literature [35], there is some uncertainty regarding the accurate detection of AC ownership, and in the disaggregation of hours into cooling and non-cooling hours. Households with very sporadic AC operation or mild AC usage within hours may have been missed by our model. Some authors suggest that hourly resolution may not be sufficient to reliably characterize AC [52]. Additionally, while our models employ household-level data for AC use and house characteristics, we use tract-level data for socioeconomic and demographic variables, which may mask individual or household-level patterns. Furthermore, while the use of a fixed CDH threshold (20.5°C) allows for comparisons between households, it assumes similar thermal responses across the population, while some households may start cooling sooner or later [37]. Qualitative research should confirm whether the differences we observe between disadvantaged and non-disadvantaged groups reflect true limitations in access or whether they constitute intentional responses to heat within comfort thresholds.

### 3.2. Policy insights

Our findings suggest that strategies seeking to promote heat adaptation and energy equity based on regional AC ownership data may be insufficient to protect the most vulnerable. Instead, AC operation metrics provide more nuanced insights about the way in which the population employs cooling, beyond having access to an AC unit. Our focus on use-based inequities (conditional on access to AC) provides a new lens for policy: affordability and operational barriers may require interventions that do not focus solely on expanding infrastructure provision (i.e., increasing access to AC systems). Rather, targeted interventions should focus on reducing the economic barriers associated with AC operation, such as energy subsidies, social tariffs, or incentives for the adoption of efficient cooling technologies. Policymakers should also consider introducing protections from utility shutoffs during hot weather, or expanding them where they exist. Currently, hot weather disconnection protections exist in only 19 U.S. states, while 42 states have cold weather protections [53]. In California, although moratoriums are currently in place for 72-hour periods with temperatures above 100°F, recent reports show spikes in electricity disconnections following the summer months [54]. Further, structural interventions, like retrofits and improvements to the building envelope in disadvantaged communities, or expanding access to solar energy, may reduce reliance on AC for cooling and help narrow existing cooling gaps.

The high temporal resolution of our dataset enabled us to model daytime and nighttime effects separately. Across meteorological, housing, and sociodemographic factors, we found effects of different magnitudes and even direction between daytime and nighttime, suggesting that different mechanisms may be operating during these times. Occupancy schedules, habits, the ability to use natural ventilation, and even electricity saving strategies (e.g., given different time of use rates) may shape cooling in different ways depending of the time of day. These differences underscore the importance of explicitly considering protections against nighttime heat extremes, a critical time for heat-related health outcomes [55, 7]. The temporal question is important as some resources, such as cooling centers (both formal and informal), may only be available during certain hours (typically during daytime).

## 4. Methods and data

### 4.1. AC operation metrics

The AC operation data are described in Peplinski et al. [39], who employed multiple linear regression to model the hourly smart-meter electricity consumption of about 200,000 households in Southern California. They first identified AC-owning households (about 140,000) based on the positive electricity-temperature relationship expected for AC [34]. Then they applied a fine-tuned algorithm to this subset to classify all hours as either electric cooling hours (*cooling state* = 1), electric heating hours (*heating state = 1*), or neither (*cooling state* and *heating state* = 0). Peplinski et al.’s algorithm accounted for hourly, weekday versus weekend, and seasonal effects (see Equation S1). In this work, we considered only those hours labeled as *cooling state* = 1, and henceforth refer to these as “cooling hours” (the remainder being “non-cooling hours”). AC (and in particular central AC) is likely the most power intensive of cooling loads, but we employ these terms to acknowledge that “cooling” might include other cooling technologies (e.g., electric fans) that contribute to the observed relationship between temperature and electricity.

We created three household-level metrics of AC operation based on Peplinski et al.’s dataset: cooling hours, cooling load, and non-cooling load. The first was a count of hours classified as *cooling state* = 1, in 12-hour intervals, resulting in measures of daytime (7 am − 6 pm in local time) and nighttime (7 pm − 6 am) cooling hours for each household. The 7-to-7 interval of hours was chosen as it maximized the number of daylight hours captured within the daytime portion throughout the year. The second and third metrics described the average hourly electricity consumed (in kWh) in cooling and non-cooling hours, respectively, also at twice-daily resolution. After cleaning, we analyzed daily AC operation data for 138,992 households in the years 2015, 2016, 2018, and 2019 (data were unavailable in 2017). We divided each year into three periods of equal duration (4 months) based on the domain-average daily mean temperature: a “hot” period running from mid-June to mid-October, where most cooling occurred; a “cool” period between December and April, with the least amount of cooling; and two “mild” periods in between the two aforementioned periods (April to mid-June and mid-October to December). These 4-month periods are illustrated in Figure 2c.

### 4.2. Supporting datasets

We obtained gridded meteorological data with hourly temporal resolution and 0.1° spatial resolution from the ERA5-Land reanalysis dataset [56]. We considered both 2-meter air temperature and relative humidity (RH; which we derive from 2-meter air temperature and wet bulb temperature) as factors affecting cooling demand following Maia-Silva et al. [57]. We resampled meteorological data to daily resolution by obtaining the daily average and maximum temperature, and the average RH. Additionally, we computed a measure of the aggregate heat burden experienced in a day by calculating the cooling degree-hours (CDH) above 20.5°C (69°F). We chose this threshold over the commonly used 18.3°C (65°F) threshold as 20.5°C was the average cooling setpoint temperature of AC users in our sample, which represents the point beyond which a positive relationship between electricity and temperature is detected. Meteorological data were assigned to census tracts based on nearest neighbors between census tract polygons and centroids of ERA5-Land grid cells.

We obtained bi-annual data relating to the socio-economic status of each census tract in the region of interest, including social, economic, and demographic dimensions, from the American Community Survey (ACS) [58] database. We considered the following variables, given previous findings in the literature associating them with heat sensitivity: economic conditions and financial burden [59, 60], literacy [21, 61], living conditions [62, 63], isolation [64], and occupational exposures [65]. We also obtained information on housing characteristics (square footage, number of bedrooms and bathrooms, year of build, and estimated value in US$) from information services provider Cotality [66]. This dataset overlapped our sample for 90,352 households (about 65% of our records). The rest of records were excluded from the analysis involving house characteristics. We computed the variable inflation factor (VIF) of all meteorological, sociodemographic, and housing-related variables and removed any variables above a VIF threshold of 5 [67]. The final set of independent variables used in this study are shown in Table S1. Additionally, building climate zone (BCZ) classifications from the California Energy Commission [68] were assigned to each census tract (see Table S2). This classification relates to differences in building codes across California, and thus considers structural differences in building practices across the region given typical meteorological conditions.

### 4.3. Statistical methods to model cooling hours and cooling load

We modeled the daily cooling hours using a two-stage (hurdle) logistic regression model, as this is a bounded, ordinal variable with discrete counts between 0 and 12 (we consider daytime and nighttime hours separately for each year). The first stage was a binary logistic regression (Equation S2), which predicted the probability of *any* cooling hours occurring for a given household-day, and given a series of independent variables (see Section 4.2). The second stage of the logistic model was an ordered logistic regression for positive cooling hour counts, which determined the probability of a number of cooling hours occurring, given some use (Equation S3). We combined the binary and ordered stages of the model to obtain the unconditional expectation of cooling hours (Equations S4 and S5). Then, we measured the effect on expected cooling hours of a change Δ*x*_*j*_ in a predictor *j*, and averaged this effect across all observations to obtain the average discrete change (ADC) for each predictor (Equation S6). Finally, we employed a Monte Carlo approach with 500 iterations to obtain the 95% confidence interval in our ADC estimates. Further details of the construction and evaluation of the logistic model can be found in Section SI.13.

We modeled the average hourly cooling and non-cooling load with ordinary least squares (OLS) regression. In order to satisfy the assumptions of OLS, we log-transformed the dependent variables *Z* (cooling or non-cooling load during daytime or nighttime), specifying them as *log*(1+ *Z*_*i*_) for each observation *i*. The linear predictor was defined as a vector of linear covariates, spline basis functions, and indicator variables (see Equation S7). We evaluated average percent changes for a discrete change Δ*x*_*j*_ in each predictor *j*, which we refer to as the average marginal effects (AME). Similarly to the logistic model, we employed a Monte Carlo approach with 500 iterations to obtain the 95% confidence interval in AME.

For both the logistic and the OLS regressions we tested versions of the models using (a) maximum daily temperature and (b) cooling degree hours (CDH). We evaluated Δ*x*_*j*_ at reasonable values for each variable, using half the standard deviation of each as a guide (see Table S1), and fitted models for daytime and nighttime hours. Model performance for the logistic model was evaluated using the Area Under the Curve (AUC) method and by plotting calibration curves. We obtained satisfactory models for both daytime and nighttime cooling hours (AUC values were 0.83 and 0.87, respectively). For the OLS models of cooling and non-cooling load, we checked that residuals were normally distributed and had low heteroskedasticity. Income effect modification was assessed by jointly testing the coefficients of the income-CDH spline interaction terms using F-tests.

### 4.4. Assessment of disparities in AC operation

We quantified inequality in cooling using Lorenz curves and Gini coefficients, which compare the cumulative share of a resource to the cumulative share of population [69]. To remove the confounding effect of heat burden, we normalized cooling hours and cooling load by the daily cumulative heat for each location, estimated using CDH above 20.5°C (see Section 4.2). We performed this normalization with daily resolution (i.e., we summed daytime and nighttime hours to compute the total cooling hours or cooling load per day). In addition, we accounted for the positive relationship between house size and AC electricity consumption (larger homes need to cool larger volumes of air) by dividing cooling load by square footage, obtaining a measure of normalized electricity intensity. We refer to these normalized metrics as ‘normalized cooling hours’ (NCH; expressed in units of hours/degree-hour) and ‘normalized cooling intensity’ (NCI; expressed in units of kWh/sq.ft./degree-hour). We employed one-tailed Mann-Whitney U tests to evaluate the differences between the lowest quintiles of cooling use and the remaining quintiles, and we tested for monotonic relationships with median income using Spearman’s rank correlation coefficient (Spearman’s *ρ*). Sample sizes correspond to the number of census tracts included in each analysis after excluding observations with missing data.

In order to assess cooling inequity, we incorporated the delineation of disadvantaged communities (DACs) by the California Environmental Protection Agency (CalEPA), following California’s State Bill 535. DACs are defined as census tracts with high pollution burden and sociodemographic characteristics that render the population highly vulnerable to hazards [48]. The locations of DACs within our study region are shown in Figure S8. In addition to considering differences by DAC status, we also compared cooling usage for different racial and ethnic groups, and across income levels. Also, we performed an analysis of spatial clusters for both normalized cooling metrics using the Local Indicators of Spatial Association (LISA) methodology [70]. LISA computes Local Moran’s *I* to identify local clusters and spatial outliers, which include *Low-Low* (low cooling tracts surrounded by other low cooling tracts), *Low-High* (low cooling tracts surrounded by high cooling tracts), *High-Low* (high cooling tracts surrounded by low cooling tracts), and *High-High* (high cooling tracts surrounded by high cooling tracts) clusters. We constructed a spatial weights matrix with *k*-nearest neighbors and a standard significance level, *α*, of 0.05. Following sensitivity testing, we chose *k* = 8 as it avoided having disconnected census tracts. We ran LISA within each BCZ, and examined the demographic breakdown of these clusters to reveal which groups cooled sparingly, and which cooled more excessively, when compared to neighboring tracts.

## Supplementary Material

This is a list of supplementary files associated with this preprint. Click to download.

- ACOperationSI.pdf

## Figures and Tables

**Table 1:** Average daily cooling hours, hourly cooling load, and hourly electricity consumption during daytime and nighttime across the four study years, split by Building Climate Zone (BCZ). N(HH) shows the number of households in each BCZ. Daytime and nighttime period are defined as hours spanning: 7 am - 6 pm and 7 pm - 6 am, respectively. See Table S2 for descriptions of BCZ.

	Daily cooling hours		Hourly cooling load (kWh)		Hourly non	cooling load (kWh)	
BCZ	*daytime*	*nighttime*	*daytime*	*nighttime*	*daytime*	*nighttime*	N(HH)
6	1.3	0.57	1.4	1.2	0.62	0.58	17,054
8	1.3	0.49	1.5	1.3	0.62	0.59	37,295
9	1.4	0.50	1.8	1.5	0.66	0.64	31,273
10	1.6	0.51	2.1	1.7	0.76	0.73	35,850
14	1.6	0.58	1.9	1.7	0.74	0.73	11,336
15	2.0	0.69	2.0	1.7	0.94	0.76	4,571
16	1.6	0.58	2.0	1.7	0.79	0.75	1,613
All	1.5	0.56	1.8	1.6	0.73	0.68	138,992

**Table 2:** Average hourly cooling load and hourly electricity consumption normalized by house square footage, split by Building Climate Zone (BCZ). N(HH) shows the number of households in each BCZ for which we have house size data (on average ~ 65% of our sample).

BCZ	Hourly cooling intensity (Wh/sq.ft.)	Hourly non-cooling intensity (Wh/sq.ft.)	N(HH)
6	0.85	0.20	8,211
8	0.95	0.21	21,764
9	1.09	0.22	20,377
10	1.10	0.22	27,118
14	1.05	0.23	8,735
15	1.17	0.26	3,078
16	1.14	0.25	1,096
All	1.05	0.23	90,352
